# Mapping Quantitative Trait Loci (QTL) in sheep. III. QTL for carcass composition traits derived from CT scans and aligned with a meta-assembly for sheep and cattle carcass QTL

**DOI:** 10.1186/1297-9686-42-36

**Published:** 2010-09-16

**Authors:** Colin R Cavanagh, Elisabeth Jonas, Matthew Hobbs, Peter C Thomson, Imke Tammen, Herman W Raadsma

**Affiliations:** 1 ReproGen - Animal Bioscience Group, Faculty of Veterinary Science, University of Sydney, 425 Werombi Road, Camden NSW 2570, Australia; 2 Food Futures National Research Flagship, CSIRO Plant Industry, Canberra, Australia

## Abstract

An (Awassi × Merino) × Merino single-sire backcross family with 165 male offspring was used to map quantitative trait loci (QTL) for body composition traits on a framework map of 189 microsatellite loci across all autosomes. Two cohorts were created from the experimental progeny to represent alternative maturity classes for body composition assessment. Animals were raised under paddock conditions prior to entering the feedlot for a 90-day fattening phase. Body composition traits were derived *in vivo *at the end of the experiment prior to slaughter at 2 (cohort 1) and 3.5 (cohort 2) years of age, using computed tomography. Image analysis was used to gain accurate predictions for 13 traits describing major fat depots, lean muscle, bone, body proportions and body weight which were used for single- and two-QTL mapping analysis. Using a maximum-likelihood approach, three highly significant (LOD ≥ 3), 15 significant (LOD ≥ 2), and 11 suggestive QTL (1.7 ≤ LOD < 2) were detected on eleven chromosomes. Regression analysis confirmed 28 of these QTL and an additional 17 suggestive (*P *< 0.1) and two significant (*P *< 0.05) QTL were identified using this method. QTL with pleiotropic effects for two or more tissues were identified on chromosomes 1, 6, 10, 14, 16 and 23. No tissue-specific QTL were identified.

A meta-assembly of ovine QTL for carcass traits from this study and public domain sources was performed and compared with a corresponding bovine meta-assembly. The assembly demonstrated QTL with effects on carcass composition in homologous regions on OAR1, 2, 6 and 21.

## Background

Sheep production is a major contributor to global food production and sheep are one of the few sources of meat with little cultural and religious restriction in consumption. Body composition traits in sheep, primarily muscle mass and fatness, are economically important to the sheep meat industry. There are numerous methods to predict body composition in sheep. Much of the variation that exists in sheep body composition is expressed as between- and within-breed differences. In order to understand the genetic architecture of these economically important traits it is essential to accurately define the phenotypes which describe carcass composition [1].

Live-weight is considered as a standard measurement of body mass, but is a poor indicator of body composition due to the inability to distinguish between different stages of physiological maturity. Body weight may be used as indicator of body composition in animals of similar genetic backgrounds and at the same physiological maturity, however, at different maturity stages the accuracy is greatly reduced [2,3]. Improved predictions of carcass composition can be determined by using ultrasound. Such scans provide a basis to estimate breeding values for eye muscle area and subcutaneous fat depth [3-5]. Increased accuracy and prediction of full body carcass characteristics can be achieved using computed tomography (CT) [6,7] but this is not routinely implemented due to cost constraints.

In addition to the difficulties in obtaining accurate carcass measurements, generation intervals are large, time to assessment is long and therefore the response to selection is slow. Therefore, the use of marker assisted selection or MAS is seen as an attractive aid to increase the efficiency of selection for these traits expensive to measure.

Linkage studies indicate the presence of one or a few major genes for increased muscling and fatness in different sheep populations [8-10]. Two full and 12 partial genome scans have reported QTL for carcass composition including bone density on chromosomes 1-6, 8, 18, 20, 21, and 24 in populations of Coopworth, Scottish Blackface, British Texel, Charollais, Suffolk, Texel and different cross-breed sheep populations [8,11-18]. At present two DNA tests (LoinMax and MyoMax; http://www.pfizeranimalgenetics.com.au/sites/PAG/aus/Pages/sheep.aspx[19]) are commercially available, which test for genetic variants in the Carwell and Myostatin genes [8,10,16,17,20-25].

This study uses CT imaging to accurately determine body composition *in vivo *in relation to body weight at two different stages of maturity. For the first time, a full genome scan was conducted to identify genomic regions associated with CT-derived parameters in an ovine backcross resource population.

## Methods

### Resource population

A resource population from crosses between fat-tail Awassi (A) and small-framed Merino (M) sheep was established. Further details of the development of the resource population can be found in Raadsma et al. [26,27]. In the QTL study reported here, only phenotypic and genotypic information from the second generation male backcross (AMM) progeny from one of four F_1 _sires was analysed in full.

### Carcass traits

The backcross progeny were weighed approximately bi-monthly until 83 weeks of age. Weights were recorded as non-fasted body weights immediately off pasture on the same day. At 83 weeks of age, male animals were randomly allocated to two management cohorts. Cohort 1 (*n *= 86) was lot fed for 90 days after which time all animals were CT scanned prior to slaughter at two years of age. Cohort 2 (*n *= 79) were grazed under paddock conditions for a further 18 months and then lot fed for 90 days followed by CT scanning and slaughter at 3.5 years of age. Both cohorts were fed *ad libitum *on a grain and lucerne pelleted ratio with a metabolisable energy content of 12.1 MJ/kg during the feedlot period. The two cohorts were created to capture the differences in fat deposition due to changes in maturity.

At the end of the *ad libitum *phase and three days prior slaughter, CT scanning was used to estimate lean, fat and bone quantities for individual sheep. Animals were fasted overnight, body weights were recorded and animals were scanned using a Hitachi CT-W400 scanner located in the Meat Science Group at the University of New England, Armidale. Animals were restrained in the supine position using three adjustable belts over the abdomen, chest and neck during the scans at 120 kV tube voltages and 150 mA current. Cross-section images were collected every 40 mm starting proximal to the *articulatio genus *(rear knee joint) and finishing at the first cervical vertebra. Between 24 and 28 images were collected from each animal depending on their length. The carcass weight was estimated from the CT images. Three sets of data (images) were derived from each image by cropping restraining equipment, internal organs and hooves, distal portion of leg, internal fat and kidney, using AUTOCAT [28]. These images provided an estimate of total body composition including hooves, internal organs and abdominal fat (first set), internal fat - comprising kidney, pelvic, mesenteric and heart fat (second set minus third set) and typical carcass components including total lean, carcass lean and total amount of bone (third set). Furthermore AUTOCAT was used to calculate the area, mean pixel value and variance of each tissue group for each animal from the three sets of images. Subcutaneous fat depth was measured over the eye muscle at the first lumbar two thirds ventral to the vertebrae. The area of fat surrounding the eye muscle (*M. longissimus dorsi*) was termed the subcutaneous fat area. The eye muscle area was estimated by averaging the area of muscle at the closest image to the first lumbar and the next caudal image. Percentages of lean, fat and bone were calculated as a percentage of the carcass weight estimated by CT (i.e. the sum of individual components estimated by CT). A list of all traits used in this study is provided in Table 1.

**Table 1:** Summary statistics of traits used in this in this study

Trait	Unit	Biological importance	*n*	AVG	SD	max	min
Body weight	kg		162	51	9.0	31	77

Carcass weight	kg		165	28	4.4	16	40

Dressing percentage	%	Proportion final weight to carcass weight	161	55	3	71	46

Total fat	kg	Indicator of total fatness	165	14	5.6	4.6	33
Carcass fat	kg	Indicator of carcass fatness	165	8.7	2.4	3.5	18
Internal fat	kg	Indicator of fatness in the internal depots	165	3.8	1.6	1.1	8.8
Percent fat in carcass	%	Proportion of fat in the carcass	165	31	4	22	45
Subcutaneous fat depth*	Pixel	Indicator of fatness	161	5.9	2.3	1	13
Subcutaneous fat area	mm^2^	Indicator of fatness	165	980	480	36	2597
Total lean	kg	Indicator of total lean	152	22	5.61	12	32
Carcass lean	kg	Indicator of muscularity	165	16	2.34	10	22
Percent lean in carcass	%	Proportion of lean in carcass	165	59	3	48	67
Eye muscle area*	mm^2^	Indicator of muscularity	165	4205	502	1245	5333
Total bone	kg	Indicator of total bone	152	7.4	4.4	2.5	12
Carcass bone	kg	Indicator of size/quantity of bone	165	2.9	0.34	1.98	4.2
Percent bone in carcass	%	Proportion of bone in carcass	165	11	2	7	16

*Industry relevant refers to a trait that is used in the industry as a standard measure and hence is incorporated as a means for comparing this study with other studies

A linear model was fitted using SAS (version 9.2) to adjust the scanning results for final body weight and cohort. For some of the traits, a scatter plot of the trait versus final body weight revealed a linear association for the first cohort but a nonlinear association for the second cohort. To allow for this nonlinearity, a quadratic term was included for the second cohort only. The full model allowing for this takes the form

$$\text{Trait}=\beta_{0}+\beta_{1}\text{Cohort2}+\beta_{2}\text{FBW}+\beta_{3}\text{Cohort2}\times \text{FBW}+\beta_{4}\text{Cohort2}\times {\text{FBW}}^{2}+\varepsilon$$

where Trait is the measurement to be adjusted for, Cohort2 is a 0-1 indicator variable taking the value 1 for the second cohort, FBW is the final body weight of the sheep, and ε is the random error. Non-significant terms from the above model were dropped, with quadratic terms retained for all traits except dressing percentage, carcass bone, percentage fat in carcass, percentage lean in carcass. Carcass weight and final body weight were adjusted only for cohort effects (Additional file 1). Residuals from the fitted models were obtained, and these were treated as the adjusted traits for subsequent QTL mapping.

### Marker analysis QTL mapping procedure

A genome scan using 189 polymorphic microsatellite markers covering all 26 sheep autosomes was conducted in 510 backcross animals. For the linkage analysis, genotypic and phenotypic information from the CT scan of 165 animals was used. The procedure of DNA extraction, genotyping, allele calling and map positions has been outlined previously [26].

QTL analyses were performed for all traits using two methods. Based on a type I error of 0.05, the design (*n *= 160 animals) had a predicted power of 0.88 to detect QTL with 0.5 SD effect [29]. Solutions were obtained using the QTL-MLE procedure for normally distributed traits in 'R' [26]. As described in previous papers [26,27], when using QTL-MLE, a QTL with LOD ≥ 3.0 was deemed highly significant, significant if LOD ≥ 2.0, and suggestive for QTL with 1.75 ≤ LOD < 2.0.

The second method involved regression analysis for a half-sib design implemented using the web-based program QTL Express [30]. QTL with chromosome-wide significance (*P *< 0.05) were described as suggestive QTL, whereas QTL exceeding the *P *< 0.01 chromosome-wide levels and *P *< 0.05 experiment-wide levels were labelled as significant and highly significant QTL, respectively. A two-QTL model was also fitted to the data using a full two-dimensional scan of each chromosome in QTL Express [30].

### Meta-assembly

A meta-assembly of QTL identified in this study was conducted by collating all known QTL from public sources for matched traits based on individual QTL locations and meta-scores as described previously [27]. The positions and confidence intervals of ovine and bovine QTL and blocks of conserved synteny across both species were identified and aligned to the genomes of both species. The individual QTL locations and their scores, and meta-score profiles can be browsed at http://crcidp.vetsci.usyd.edu.au/cgi-bin/gbrowse/oaries_genome/. In addition to the lactation traits, QTL profiles for growth, body weight and carcass composition can now be browsed on this website. Growth and body weight meta-scores from the first paper of this series [26] were also loaded into the website. The carcass composition traits were summarised into four trait classes: bone (percentage bone, bone weight, bone yield), fat (fat yield, back fat, fat depth, marbling, fat thickness, subcutaneous fat thickness), muscle (longissimus muscle area, rib eye area, carcass yield, retail product yield, shear force, lean meat yield) and weight (hot and cold carcass weight, yearling, weaning and slaughter weight). Single and aggregated bars, heat maps and plots can be selected for sheep and cattle as well as meta-scores for both species. Hyperlinks to the original manuscript reference are given.

## Results

### Analysis of carcass data

The summary statistics for each phenotype are shown in Table 1. For the second cohort, carcass weight and predicted carcass weight from the scan were highly correlated (*r *= 0.90, *P *< 0.01) and both traits were also highly correlated with final body weight (*r *= 0.92 and 0.89, for both cohorts respectively, *P *< 0.01) (Additional file 2). Across both cohorts, the average body weight at scanning was 51 kg, with an average carcass weight of 28 kg (dressing percentage 55%). Animals from cohort 2 were significantly (*P *< 0.01) heavier, with a higher mass of total bone, fat and lean compared to cohort 1. However, they had a significantly (*P *< 0.01) lower percentage bone in the carcass (Additional file 3). Within tissue groups, lean, fat (except internal fat and subcutaneous fat depth) and bone parameters were significantly correlated (*r *= 0.27 to 0.81, all *P *< 0.01) (Additional file 4). Significant correlations (*P *< 0.05) were also detected between many traits among fat and lean tissue groups, with the highest correlation between percentage lean and fat (*r *= -0.97, *P *< 0.01). No significant correlations were detected between carcass bone, total bone and eye muscle area and most of the other traits (Additional file 4).

### Putative QTL identified

**Figure 1:**
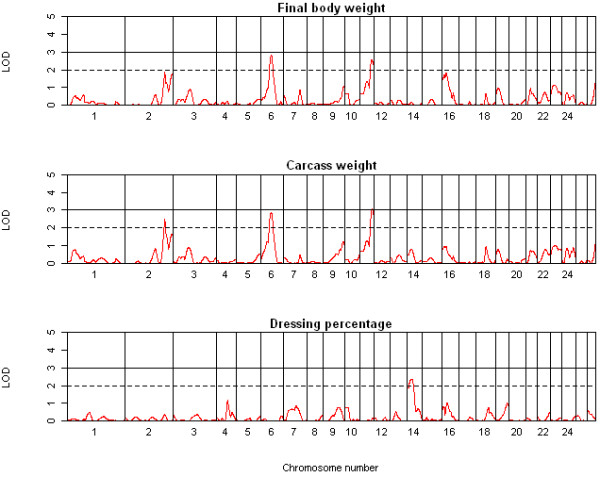
**QTL map of the entire genome for body and carcass weight and dressing percentage**.

**Figure 2:**
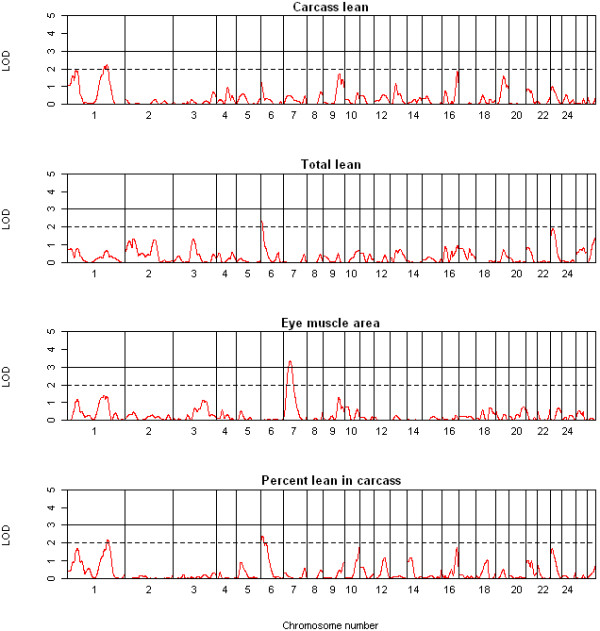
**QTL map of the entire genome for carcass lean, total lean, eye muscle area and lean percentage**.

**Figure 3:**
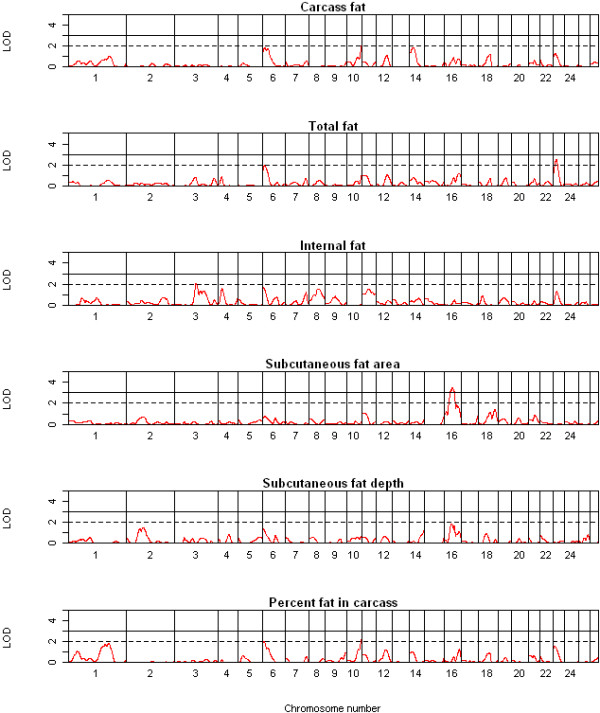
**QTL map of the entire genome for carcass fat, total fat, internal fat, subcutaneous fat depth, subcutaneous fat area and percentage fat**.

**Figure 4:**
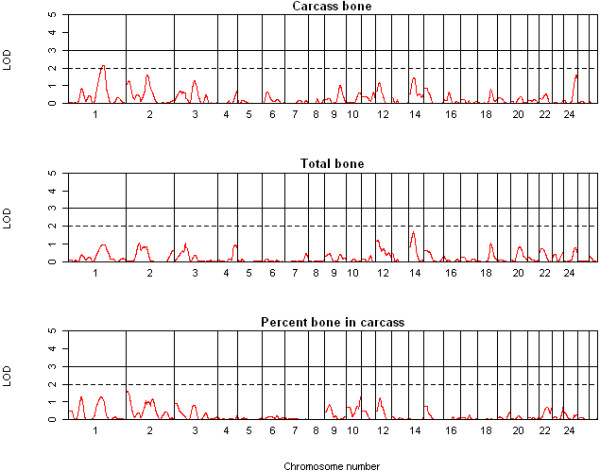
**QTL map of the entire genome for total bone, carcass bone and bone percentage**.

In total, three highly significant (LOD ≥ 3.0), 15 significant (LOD ≥ 2.0) and 12 suggestive (1.7 < LOD < 2.0) QTL were detected on chromosomes 1 to 3, 6, 7, 9-11, 14, 16 and 23 across the 13 traits using QTL-MLE. A summary of the suggestive and significant QTL positions, effect sizes, and 1-LOD support intervals is shown in Table 2. The genome-wide LOD score profiles for all traits are shown in Figures 1, 2, 3 and 4. With the exception of one suggestive QTL on chromosome 6, all QTL detected by QTL-MLE were confirmed by the QTL regression analysis of QTL Express. A total of five highly significant (experiment-wide *P *< 0.05), six significant (chromosome-wide *P *< 0.01) and 34 suggestive (chromosome-wide *P *< 0.05) QTL were identified on chromosomes 1-3, 6, 7, 9, 10, 11, 14, 16, 19, 23 and 26 using QTL Express (Additional file 5). Among these, two significant (chromosome-wide *P *< 0.01) and 16 suggestive (chromosome-wide *P *< 0.05) QTL on chromosomes 6, 8-14, 16, 23 and 26 were not detected using QTL-MLE. Confidence intervals and 1-LOD support intervals for QTL locations extended across a large proportion of each of the chromosomes (Table 2, additional file 5).

**Table 2:** Summary of QTL for carcass traits using QTL-MLE

OAR	Trait	QTL [cM]	1-LOD support interval [cM]	Marker closest to peak	Lower marker	Upper marker	LOD score	QTL effect(SD)
1	Carcass bone	261	220 - 277	CSSM4	MAF64	INRA011	2.1**	0.56
1	Carcass lean	293	238 - 314	INRA011	CSSM4	BM6506	2.2**	0.69
1	Percent fat in carcass	296	228 - 324	INRA011	CSSM4	BM6506	1.8*	-0.60
1	Percent lean in carcass	299	253 - 323	BM6506	INRA011	BMS4045	2.2**	0.68

2	Carcass weight	294	284 - 309	MCM554	CSSM045	ARO28	2.5**	0.60
2	Final body weight	294	280 - 318	MCM554	CSSM045	ARO28	1.9*	0.51

3	Internal fat	155	144 - 175	BM827	BM304	EPCDV25	2.1**	0.57

6	Internal fat	8	5 - 32	OARCP125	OARCP125	MCM204	1.7*	0.50
6	Percent fat in carcass	10	5 - 50	OARCP125	OARCP125	MCM204	2.0**	0.57
6	Percent lean in carcass	13	5 - 52	OARCP125	OARCP125	BM1329	2.4**	-0.64
6	Total fat	15	5 - 42	OARCP125	OARCP125	BM1329	2.0**	0.61
6	Carcass fat	16	5 - 61	OARCP125	OARCP125	BM1329	1.8*	0.56
6	Carcass weight	75	60 - 91	OARHH55	BM1329	OARJMP1	2.8**	0.64
6	Final body weight	76	62 - 91	OARHH55	BM1329	OARJMP1	2.8**	0.64

7	Eye muscle area	51	29 - 70	BMS528	BM3033	MCM223	3.4***	-0.99

9	Carcass lean	116	95 - 154	BMS1304	MAF33	BM4513	1.7*	0.51

10	Carcass fat	112	101 - 112	OARDB3	TGLA441	OARDB3	2.1**	0.68
10	Percent fat in carcass	112	98 - 112	OARDB3	TGLA441	OARDB3	2.3**	0.71
10	Percent lean in carcass	112	81 - 112	OARDB3	TGLA441	OARDB3	1.8*	-0.62

11	Carcass weight	92	79 - 107	EPCDV23	BM17132	ETH3	3.1***	0.64
11	Final body weight	88	75 - 107	EPCDV23	BM17132	ETH3	2.5**	0.62

14	Carcass fat	29	14 - 54	CSRD270	TGLA357	MCM133	1.8*	-0.53
14	Dressing percentage	33	14 - 56	CSRD270	TGLA357	MCM133	2.38**	-0.57
14	Total bone	36	14 - 57	CSRD270	TGLA357	MCM133	1.7*	-0.47

16	Final body weight	32	1 - 60	OARCP99	BM1225	TGLA126	1.8*	-0.58
16	Percent lean in carcass	113	95 - 121	MCM150	DIK4612	DIK2269	1.8*	-0.48
16	Subcutaneous fat area	62	38 - 75	BMS2361	TGLA126	BM4107	3.5***	0.73

23	Percent lean in carcass	14	3 - 45	MCMA1	BL006	MAF35	1.7*	0.57
23	Total fat	25	8 - 38	MCMA1	BL006	MAF35	2.5**	-0.61

Shown are the relative positions and the confidence interval (CI) along the 1 male distance map [26], *P*-values were obtained from likelihood ratio tests (LRT) with 1 df (QTL only); * 1.75 ≤ LOD < 2.0, ** 2.0 ≤ LOD < 3.0, *** LOD ≥ 3.0; standardised QTL effects (SD) are expressed as the estimated effect difference (Awassi - Merino) relative to the estimated residual standard deviation

Common QTL for body and carcass weight were identified on chromosomes 2, 6 and 11 using both QTL analysis methods, in addition to the QTL for body weight on chromosome 16 and for dressing percentage on chromosome 14. For muscle traits, eight QTL were detected on seven chromosomes, for fat traits ten QTL on seven chromosomes and for bone traits only two QTL. There were no QTL which solely contributed to traits related to a single tissue i.e. QTL just for muscle, fat or bone. For chromosomes 1, 6, 10, 14, 16 and 23, the QTL for different tissue groups acted pleiotropically, with the same QTL describing traits for different tissue groups. Among the six QTL identified on chromosome 6, two were for weight and three for fat parameters, although the peak positions of the QTL for these two traits groups differed. Similarly, the QTL regions for final body weight, percent lean and subcutaneous fat area were all on chromosome 16, but the peak positions varied. The effect sizes of the QTL ranged from 0.73 to 0.99 SD (Table 2) and accounted for 3.8 to 9.4% of the phenotypic variance (Additional file 5). Three of the QTL identified here were deemed cryptic QTL, with an effect opposite to what was expected based on breed of origin.

The two-QTL model implemented in QTL Express showed four pairs of QTL which were separated by at least one marker; carcass lean (OAR1), percent bone (OAR1), percent fat (OAR18) and internal fat (OAR19). QTL for carcass lean on chromosome 1 were in coupling phase, whereas all other QTL pairs were in repulsion phase. The QTL in repulsion phase were not identified using the single QTL model since the opposite sign of the QTL effects may have prevented detection under the single QTL model. Details describing QTL positions and effect sizes, and comparisons with single and no QTL models are in Table 3.

**Table 3:** Summary of significant QTL for carcass traits using QTL Express under a two-QTL model

OAR	Trait	Position QTL [cM] with flanking markers		*F*-value		**Herit [%]**^**4**^	**QTL effect SD (SE)**^**3**^	
		A	B	**2 vs 0**^**1**^	**2 vs 1**^**2**^		A	B
1	Carcass lean	40 BMS835-OARHH51	272 INRA011-BM6506	9.4*	8.7*	9.5	0.642 (0.218)	0.803 (0.258)
1	Percent bone in carcass	72 OARHH51-BM6465	216 MAF64-CSSM4	6.8*	7.3*	6.9	-74.3 (26.5)	102.2 (37.6)

9	Eye muscle area	72 ILST011-MAF33	76 MAF33-BMS1304	6.8*	6.8*	6.8	-0.0198 (0.0054)	0.0207 (0.0057)

18	Percent fat in carcass	80 BM7243-OARHH47	88 TGLA122-MCM38	6.0	8.1*	5.9	62.6 (18.2)	-55.7 (18.2)

19	Internal fat	80 OAFCB304-MCM111	88 MCM111-OARCP88	7.1*	11**	7.1	-3.54 (0.94)	3.35 (0.92)

^1^*F*(2 versus 0) is *F*-statistic for testing two QTL vs no QTL on chromosome

^2^*F*(2 versus 1) is *F*-statistic for testing two QTL vs one QTL on chromosome

^3^standardised QTL effect (SD) = QTL Effect/Residual Std Dev; and the standard error (SE) of QTL positions A and B

^4^variance or QTL heritability as a proportion of the phenotypic variance accounted for by the QTL in %

* chromosome-wide *P *< 0.05; ** chromosome-wide *P *< 0.01

### Meta-assembly

**Figure 5:**
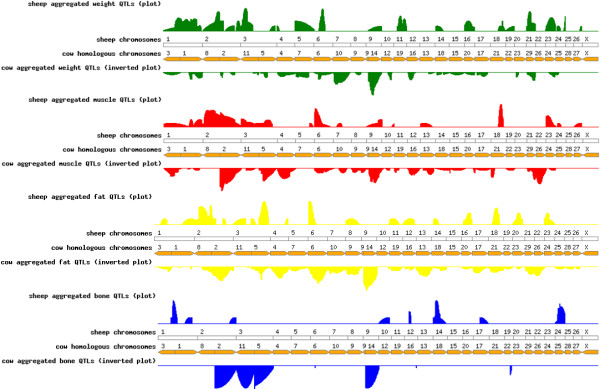
**Comparative genome map of aggregated meta-scores for carcass-related QTL derived from sheep and cattle studies**.

Published QTL reports for carcass traits in sheep, comprising four genome-wide linkage studies [26,31-33] and 13 partial genome scans [8,11,13-18,34-36] were used for the meta-assembly. QTL for a wide range of carcass traits, including traits not measured in our study (muscle growth, muscle depth, and meat colour), were reported on chromosomes 1-6, 8, 11, 18, 20, 21, 23, 24 and 26 in various sheep populations [8,13,15-18,31-33,35,36]. For two of the studies, the locations of the QTL were not given [11,34]. No QTL were reported on chromosomes 7, 9, 10, 12-17, 19, 22, and 25, but these results might be biased due to partial genome scans, favouring chromosomes with known QTL or candidate genes. The meta-scores showed consistency on six regions of interest across multiple studies for fat, muscle and weight traits, specifically for fat on OAR2 (BTA2) and OAR6 (BTA6), for muscle QTL on OAR2 (BTA2) and for weight on OAR1 (BTA1), 6 (BTA6) and 21 (BTA29) (Figure 5).

The results of the ovine and bovine meta-assembly are shown as a comparative meta-score plot against the ovine genome in Figure 5 and are visualised on the ovine genome browser http://crcidp.vetsci.usyd.edu.au/cgi-bin/gbrowse/oaries_genome/. The very broad range of traits describing carcass and body composition in cattle resulted in QTL being reported on all chromosomes. Furthermore, in contrast to studies in sheep, the majority of studies in cattle reviewed here refer to genome-wide genome scans (*n *= 14) [37-39]. In addition, eight partial genome scans or candidate gene analyses in cattle were included here [40-47].

## Discussion

This study is interesting in that it is the fourth full genome scan for mapping QTL in sheep with respect to carcass traits, and the first where carcass traits were determined from data derived by CT scan which can provide highly accurate profiles of tissue distribution.

### Analysis of carcass data

CT scanning was first developed for medical applications and has been extended to animal applications since the 1980s, firstly in pigs and subsequently in sheep [48]. Experiments in sheep and lambs showed that the correlation between CT measures of carcass composition and those derived from manual dissection is very high, but CT or virtual dissection is more precise and reliable [48]. Our study confirmed the high correlation between carcass weight and estimates of carcass weight from scanning [49]. Compared to ultrasound, the standard errors of the predicted values are lower [48,50]. Vester-Christensen et al. [51] and Young et al. [48] have proposed that CT scanning should be an essential reference tool for body and carcass composition. The use of the more precise phenotypes derived from CT measures will also lead to better phenotypes for genetic analysis. Heritabilities for CT-derived traits have been found to be moderate to high [48,52,53]. Theoretical predictions of the genetic progress by incorporating CT traits into selection indices suggest increases in response by 50% or even 100% when combining different measurement methods [6].

The sheep in our study were managed as two cohorts. These cohorts differed significantly in carcass weight and stage of maturity and were considerably heavier than animals in studies published previously [49]. Animals investigated here were taken to a greater stage of maturity to measure specific effects on fat and fat distribution. Sheep from cohort 1 had similar muscle/carcass lean weights compared to meat sheep [54] and Norwegian lambs [49]. However, for both these studies, the proportion of muscle was higher than in our study, largely due to differences in fatness and stage of development (age, maturity). For the same reasons, the proportion of bone in the carcass was lower in our study than in studies presented by Young et al. [54] and Kongsro et al. [49].

The main focus of our project was the study of fat characteristics in the carcass. Therefore, older and consequently more mature sheep were used. Adjusting body composition traits for body weight at the time of scanning was considered the best method to accurately measure tissue groups independently of their body mass. Animals from the second cohort had higher fat content and total percent fat compared to animals from cohort 1. There were significant correlations between the major tissue groups (lean, fat and bone). Fat traits tended to be significantly and negatively correlated with lean traits, as reported by Lambe et al. [55]. Without adjusting for body weight, the correlations would have been strongly positive [55,56], as was also the case here (results not shown). The importance of adjustment for body weight is that properties of body tissue can be investigated free from the effects of body mass. The differences in stage of maturity resulted in different adjustments for body weight, namely a linear effect for cohort 1 and a curvilinear effect for cohort 2, suggesting a plateau of growth had been reached and animals were in the mature fattening phase of development.

### QTL analysis

Genome-wise error rates were controlled by adjustment of *P*-values through the use of a chromosome- and experiment-wide permutation test in the case of QTL Express, therefore the number of false positive QTL was assumed to be minimal. For the maximum-likelihood analysis we chose thresholds for a LOD statistic which was deemed to be conservative at LOD of 2 (*P *≈ 0.01) and LOD of 3 (*P *≈ 0.001). The close agreement between the number of QTL detected in each method suggests that the likelihood of random false positives is expected to be small.

For body and carcass weight, QTL were identified on chromosomes 2, 6, 11 and 16. The QTL on chromosomes 6 and 11 were consistent with those reported in the same study population at earlier time points [26]. The QTL for final and carcass weight on chromosome 2 was the only one that corresponded to a QTL for live weight in Scottish Blackface and Suffolk, Texel sheep [13,17]. A total of eight QTL across seven chromosomal regions were identified for muscle. QTL on chromosomes 1 and 6 were consistent with other studies in Suffolk and Texel populations [11,16,17], whereas QTL on chromosomes 7, 9, 10, 16 and 23 can be considered novel.

QTL for fat have previously been reported on OAR 1-4, 18 and 20 in different sheep populations [14,16,17,31,33,34]. Within the confidence interval of our QTL, we confirmed QTL on chromosome 1 and 3, and novel QTL were identified on OAR 6, 10, 14, 16 and 23. QTL for fatness have consistently been reported on chromosomes 2, 3 and 18 [14,16,17], but the QTL on OAR18 was only identified using the two-QTL model and no QTL on OAR2 was detected in the current study despite the emphasis on fat traits.

Few reports are available for bone-related traits in sheep, and no QTL study on bone yield in the carcass has been reported. Previous QTL studies have analysed bone density and cross sectional area in Scottish Blackface and Coopworth sheep [13,31,32]. The two QTL detected here for bone yield suggest that the QTL landscape is rather featureless for this trait.

In summary, the first interesting discovery of this paper was the identification of novel QTL with small to moderate effects on body composition and body weight on chromosomes 1, 6, 7, 9, 10, 14, 16 and 23. This may in part be due to an increase in accuracy of phenotyping using CT image analysis.

A notable finding of this study was that there were no QTL which exclusively affected multiple measures of the same tissue group, i.e. fat, lean or bone. The effect of measuring fat at individual or a limited number of sites was discussed by Thompson [57], who proposed that individual depots may not reflect total body fat in the animal. This is seen in the correlations of non-unity between traits indicative of fatness at different sites (Table 1). This suggests that different measures of the same tissue reflect different traits with different QTL. This may have implications for QTL detection and application. For instance, QTL used to reduce subcutaneous fat content may not necessarily result in a reduction of total carcass fat.

Despite many QTL reports and a significant association on chromosome 18, we could not confirm the effects of the important loci such as the Carwell and Callipyge genes with known effects on muscle lean in sheep [20,58,59]. These and other genes on the same chromosomal region are known to be imprinted with paternally expressed protein-coding genes, as well as several maternally expressed non-coding RNA genes [20], which may have prevented their detection in our study, which used only one paternal half-sib family. Single-marker association analyses revealed significant associations for markers close these genes (results not shown) but this requires more detailed analysis. In future studies, the use of multi-sire families and linkage disequilibrium among maternal alleles should focus on the identification of these imprinting effects.

We considered an interdependency of QTL for body weight on OAR11 and 16. These chromosomes contain the growth hormone (GH) and growth hormone receptor (GHR) genes, with known effects on body weight and carcass composition across species [60-66]. Even though we identified QTL for final body weight on both chromosomes, we could not detect an interaction between these two QTL and assume that the genes underlying these two QTL act in a simple additive fashion.

Although we examined the likely importance of two QTL for all traits and report on five cases with significant support for QTL pairs, the interpretation of the results warrant caution, especially where the QTL are closely located or no corresponding QTL were detected under the single QTL model.

### Meta-assembly and comparative analysis

A meta-assembly of QTL identified for carcass traits was conducted by collating all known ovine QTL from public sources for matched traits, as previously described [27]. Additionally, studies in cattle were summarised using the same methodology. A summary of the carcass meta-scores from cattle and sheep that is incorporated into the ovine genome browser http://crcidp.vetsci.usyd.edu.au/cgi-bin/gbrowse/oaries_genome/ is shown in Figure 5. Furthermore, due to the lack of studies based on CT-derived phenotypes, and the different methods, models, and population types used across studies, we considered them to be the same carcass trait if they described the same carcass characteristic.

Despite the large number of QTL detected in cattle, relatively few QTL in sheep were found in comparative locations. However, for some traits, especially for muscle and fat, loci were mapped to homologous regions; these QTL may possibly describe the same gene.

Among the 11 studies summarised using the meta-assembly in cattle, four reported QTL for body weight on chromosome 14 [39,46,67,68], but no QTL was found in sheep in the homologous position. The highest ovine meta-scores for body weight were derived for chromosomes 1 [13,14,17], 6 [26] and 21 [13,26]. QTL were reported for body weight in cattle on the homologous chromosomes 1 [37,39,67], 6 [40,67,69] and 29 [39,70,71].

Amongst the six ovine and 13 bovine QTL studies reporting linkage regions for muscle related traits, we found one region in common between sheep and cattle, namely OAR2 and BTA2 for carcass weight, eye muscle area and retail product yield [8,16,36-38,67,70,72] (Figure 5).

High meta-scores for fat QTL were derived for sheep and cattle on homologous chromosomes OAR2/BTA2 and OAR6/BTA6 [37,38,45,67,68,70,71,73]. However, no QTL for fat traits were identified in sheep, which align to bovine chromosome 14, where the highest meta-score was calculated for corresponding traits in cattle [46,67,69,70,74].

No homologous regions were found between sheep and cattle for bone traits. This is likely due to the limited number of studies conducted and QTL reported for these traits in either species.

## Conclusion

This is the first study using CT-derived carcass measures for a full-genome scan in sheep. To our knowledge this is the only study with a focus on carcass fat characteristics in mature sheep. We present evidence for a significant number of new QTL for muscle, fat and bone traits in sheep. We also confirm and support the presence of previously published QTL in breeds other than those studied here. Finally, homology in QTL regions between sheep and cattle for muscle, bone, fat and body weight was demonstrated.

## Competing interests

The authors declare that they have no competing interests.

## Authors' contributions

CRC analysed the CT scans of the second cohort and ran the QTL analyses, was responsible for the data assembly, phenotypic analysis, contributed to genotyping and preparing the manuscript. EJ ran the final models and QTL analyses, participated in the manuscript preparation. MH calculated the QTL scores and designed the online data source. PCT developed the QTL methodology, implemented the QTL-MLE program, and contributed to manuscript preparation and the overall design. IT was involved in the project management and participated in the preparation of the manuscript, HWR was responsible for the overall design, project management, and was involved in analysis of the data and writing the manuscript. All authors read and approved the final manuscript.

## Supplementary Material

Additional file 1 **Summary of linear models for trait pre-correction used in this study**. *R*-square, overall *P*-value (Model *P*-value) and *P*-values for the regression coefficients in the fitted models. Models were adjusted for final body weight (FBW) and cohort allowing for nonlinearity if observed in a scatter plot of the trait versus final body weight Click here for file

Additional file 2 **Phenotypic correlation between body weight and carcass weight measures**. Phenotypic correlation between body weight and carcass weight measures. Correlations exceeded the *P *< 0.01 threshold using with *n *= 160 animals and ^1^*n *= 72 animals Click here for file

Additional file 3 **Summary statistics of traits used in this study**. Summary statistics of traits used in this in this study within the two cohorts Click here for file

Additional file 4 **Phenotypic correlation between the carcass traits**. Phenotypic correlation between carcass lean (CL), total lean (TL), eye muscle area (EMA), carcass fat (CF), total fat (TF), internal fat (IF), subcutaneous fat depth (SFD), carcass bone (B), total bone (TB), percent lean in carcass (PL), percent fat in carcass (PF), percent bone in carcass (PB); *r *> 0.2 corresponds to *P *< 0.05, and *r *> 0.3 corresponds to *P *< 0.01 with *n *= 160 Click here for file

Additional file 5 **QTL for body weight and carcass traits using QTL Express**. QTL for body weight and carcass traits using QTL Express; *chromosome-wide *P *< 0.05; **chromosome-wide *P *< 0.01; ***experiment-wide *P *< 0.05; ****experiment-wide *P *< 0.01; variance or QTL heritability (Heritab) as a proportion of the phenotypic variance accounted for by the QTL [variance explained by the QTL effect = 1-(MS of full model/MS of reduced model)] Click here for file
