# Supplementary material for: Mapping Quantitative Trait Loci (QTL) in sheep. III. QTL for carcass composition traits derived from CT scans and aligned with a meta-assembly for sheep and cattle carcass QTL
Source: Genet Sel Evol. 2010 Sep 16;42(1):36. doi: 10.1186/1297-9686-42-36 (PMC2949606; doi:10.1186/1297-9686-42-36)
Supplement: Additional file 1 — Summary of linear models for trait pre-correction used in this study. R-square, overall P-value (Model P-value) and P-values for the regression coefficients in the fitted models. Models were adjusted for final body weight (FBW) and cohort allowing for nonlinearity if observed in a scatter plot of the trait versus final body weight [file 1297-9686-42-36-S1.PDF]

Additional file 1 - Summary of linear models for trait pre-correction used in this study

Trait	*R*-square	Model *P*-value	Cohort2	FBW	Cohort2× FBW	Cohort2× FBW^2
Carcass lean	0.86	<.0001	0.0018	<.0001	0.0002	<.0001
Carcass weight	0.36	<.0001	<.0001			
Final body weight	0.52	<.0001	<.0001			
Dressing percentage	0.38	<.0001	<.0001			
Carcass bone	0.63	<.0001	<.0001	<.0001		
Total fat	0.92	<.0001	0.9345	0.0172	0.8432	0.2695
Subcutaneous fat area	0.62	<.0001	0.5296	0.6349	0.3897	0.2351
Total lean	0.94	<.0001	0.0926	<.0001	0.0024	0.0010
Eye muscle area	0.51	<.0001	0.0464	<.0001		<.0001
Carcass fat	0.83	<.0001	0.7502	0.0515	0.3088	0.3088
Internal fat	0.67	<.0001	0.9420	0.2108	0.8426	0.5509
Subcutaneous fat depth	0.35	<.0001	0.8971	0.6743	0.8076	0.7350
Total bone	0.997	<.0001	0.0076	<.0001	<.0001	<.0001
Percentage fat in carcass	0.51	<.0001	0.0046	<.0001	0.0334	
Percentage lean in carcass	0.41	<.0001	<.0001	<.0001	0.0072	
Percentage bone in carcass	0.87	<.0001	0.7372	0.0013	0.2184	0.2184
