# Supplementary material for: Mapping Quantitative Trait Loci (QTL) in sheep. III. QTL for carcass composition traits derived from CT scans and aligned with a meta-assembly for sheep and cattle carcass QTL
Source: Genet Sel Evol. 2010 Sep 16;42(1):36. doi: 10.1186/1297-9686-42-36 (PMC2949606; doi:10.1186/1297-9686-42-36)
Supplement: Additional file 2 — Phenotypic correlation between body weight and carcass weight measures. Phenotypic correlation between body weight and carcass weight measures. Correlations exceeded the P < 0.01 threshold using with n = 160 animals and 1n = 72 animals [file 1297-9686-42-36-S2.PDF]

Additional file 2 - Phenotypic correlation between body weight and carcass weight measures

Trait	Final weight	Carcass weight
Carcass weight	0.92	
Carcass weight (Scan)[1]	0.89	0.90
