# Supplementary material for: Mapping Quantitative Trait Loci (QTL) in sheep. III. QTL for carcass composition traits derived from CT scans and aligned with a meta-assembly for sheep and cattle carcass QTL
Source: Genet Sel Evol. 2010 Sep 16;42(1):36. doi: 10.1186/1297-9686-42-36 (PMC2949606; doi:10.1186/1297-9686-42-36)
Supplement: Additional file 3 — Summary statistics of traits used in this study. Summary statistics of traits used in this in this study within the two cohorts [file 1297-9686-42-36-S3.PDF]

Additional file 3 - Summary statistics of traits used in this study

Trait	Cohort	*n*	AVG	SD	max	min
Body weight end	All	162	51	9.0	31	77
	1	85	45	4.9	31	60
	2	77	58	7.5	34	77
Carcass weight	All	165	28	4.4	16	40
	1	86	25	2.9	16	34
	2	79	30	4.2	17	40
Dressing percentage	All	161	0.55	0.03	0.71	0.46
	1	84	0.57	0.03	0.71	0.46
	2	77	0.53	0.02	0.57	0.48
Total fat	All	165	14	5.6	4.6	33
	1	86	10	2.1	4.6	18
	2	79	19	4.5	7.9	33
Carcass fat	All	165	8.7	2.4	3.5	18
	1	86	7.6	1.4	3.5	12
	2	79	10	2.6	4.1	18
Internal fat	All	165	3.8	1.6	1.1	8.8
	1	86	2.9	0.93	1.1	6.8
	2	79	4.7	1.6	1.3	8.8
Percent fat in carcass	All	165	0.31	0.04	0.22	0.45
	1	86	0.29	0.03	0.22	0.36
	2	79	0.32	0.04	0.23	0.45
Subcutaneous fat depth	All	161	5.9	2.3	1	13
	1	85	5.1	2.0	1	11
	2	76	6.8	2.3	2.5	13
Subcutaneous fat area	All	165	980	480	36	2597
	1	86	725	312	128	1736
	2	79	1257	478	35	2597
Total lean	All	152	22	5.61	12	32
	1	79	17	1.84	12	25
	2	73	27	2.6	19	32.
Carcass lean	All	165	16	2.34	10	22
	1	86	14	1.61	10	21
	2	79	18	1.81	11	22
Percent lean in carcass	All	165	0.59	0.03	0.48	0.67
	1	86	0.58	0.03	0.52	0.64
	2	79	0.59	0.04	0.48	0.67
Eye muscle area	All	165	4205	502	1245	5333
	1	86	4140	452	2724	5076
	2	79	4276	545	1245	5333
Total bone	All	152	7.4	4.4	2.5	12
	1	79	3.1	0.3	2.5	4.3
	2	73	12	0.45	10	12
Carcass bone	All	165	2.9	0.34	1.98	4.2
	1	86	3.12	0.29	2.53	4.23
	2	79	2.70	0.25	1.98	3.23
Percent bone in carcass	All	165	0.11	0.02	0.07	0.16
	1	86	0.12	0.01	0.10	0.16
	2	79	0.09	0.01	0.07	0.11
