# Supplementary material for: Mapping Quantitative Trait Loci (QTL) in sheep. III. QTL for carcass composition traits derived from CT scans and aligned with a meta-assembly for sheep and cattle carcass QTL
Source: Genet Sel Evol. 2010 Sep 16;42(1):36. doi: 10.1186/1297-9686-42-36 (PMC2949606; doi:10.1186/1297-9686-42-36)
Supplement: Additional file 4 — Phenotypic correlation between the carcass traits. Phenotypic correlation between carcass lean (CL), total lean (TL), eye muscle area (EMA), carcass fat (CF), total fat (TF), internal fat (IF), subcutaneous fat depth (SFD), carcass bone (B), total bone (TB), percent lean in carcass (PL), percent fat in carcass (PF), percent bone in carcass (PB); r > 0.2 corresponds to P < 0.05, and r > 0.3 corresponds to P < 0.01 with n = 160 [file 1297-9686-42-36-S4.PDF]

Additional file 4: Phenotypic correlation between the carcass traits

Carcass lean	CL	TL	EMA	CF	TF	IF	SFA	SFD	B	TB	PL	PF
Total lean	0.75											
Eye muscle area	0.56	0.34										
Carcass fat	-0.29	-0.37	-0.17									
Total fat	-0.37	-0.53	-0.15	0.81								
Internal fat	-0.26	-0.33	-0.09	0.36	0.72							
Subcutaneous fat depth	-0.29	-0.29	-0.17	0.49	0.38	0.15						
Subcutaneous fat area	-0.28	-0.30	-0.06	0.61	0.53	0.27	0.79					
Carcass bone	0.41	0.32	0.19	-0.05	-0.15	-0.22	-0.03	-0.06				
Total bone	0.41	0.33	0.15	0.03	-0.12	-0.18	0.03	-0.01	0.81			
Percent lean in carcass	0.64	0.59	0.39	-0.89	-0.77	-0.36	-0.52	-0.59	0.09	0.04		
Percent fat in carcass	-0.59	-0.56	-0.34	0.92	0.80	0.40	0.52	0.61	-0.26	-0.18	-0.97	
Percent bone in carcass	0.02	0.10	-0.05	-0.44	-0.40	-0.27	-0.17	-0.27	0.71	0.56	0.25	-0.47
