# Supplementary material for: Mapping Quantitative Trait Loci (QTL) in sheep. III. QTL for carcass composition traits derived from CT scans and aligned with a meta-assembly for sheep and cattle carcass QTL
Source: Genet Sel Evol. 2010 Sep 16;42(1):36. doi: 10.1186/1297-9686-42-36 (PMC2949606; doi:10.1186/1297-9686-42-36)
Supplement: Additional file 5 — QTL for body weight and carcass traits using QTL Express. QTL for body weight and carcass traits using QTL Express; *chromosome-wide P < 0.05; **chromosome-wide P < 0.01; ***experiment-wide P < 0.05; ****experiment-wide P < 0.01; variance or QTL heritability (Heritab) as a proportion of the phenotypic variance accounted for by the QTL [variance explained by the QTL effect = 1-(MS of full model/MS of reduced model)] [file 1297-9686-42-36-S5.PDF]

Additional file 5: QTL for body weight and carcass traits using QTL Express

OAR	Trait	Position [cM]	CI [cM]	*F*-Value	Heritab [%]
1	Carcass bone	220	60 - 356	8.86*	4.68
1	Carcass lean	272	4 - 292	9.59*	5.10
1	Percent fat in carcass	276	24 - 400	9.12*	4.77
1	Percent lean in carcass	280	14 - 308	10.9*	5.86
2	Carcass weight	292	100 - 356	10.71*	5.72
2	Final body weight	292	60 - 356	8.56*	4.51
3	Internal fat	156	80 - 262	8.5*	4.48
6	Carcass fat	12	0 - 160	7.42*	3.86
6	Carcass weight	72	28 - 96	13.76***	7.38
6	Final body weight	72	6 - 140	13.29***	7.13
6	Percent fat in carcass	8	0 - 160	7.88*	4.12
6	Percent lean in carcass	12	0 - 152	9.62*	5.07
6	Total fat	12	0 - 160	7.72*	4.03
6	Total lean	0	0 - 124	9.36*	4.96
7	Eye muscle area	56	20 - 96	13.59***	7.30
8	Internal fat	44	0 - 104	7.22*	3.74
9	Carcass lean	112	0 - 144	8.5*	4.48
9	Carcass weight	136	16 - 144	6.68*	3.43
9	Eye muscle area	104	0 - 144	6.66*	3.41
10	Carcass fat	84	0 - 84	9.1*	4.82
10	Percent bone in carcass	84	0 - 84	7.18*	3.45
10	Percent fat in carcass	84	0 - 84	10.52**	5.59
10	Percent lean in carcass	84	0 - 84	8.19*	4.28
11	Carcass weight	68	12 - 76	15.51***	8.31
11	Final body weight	68	8 - 76	12.19**	6.54
11	Internal fat	28	4 - 76	9.32**	4.94
12	Percent fat in carcass	60	0 - 92	6.94*	3.54
12	Percent lean in carcass	68	0 - 88	6.64*	3.40
13	Carcass lean	32	0 - 96	6.61*	3.39
14	Carcass bone	24	0 - 100	6.47*	3.31
14	Carcass fat	16	0 - 96	9.21**	4.88
14	Dressing percentage	16	4 - 62	12.52**	6.75
14	Total bone	20	4 - 88	7.25*	3.76
16	Dressing percentage	28	4 - 124	6.81*	3.51
16	Final body weight	16	0 - 84	9.02*	4.77
16	Percent lean in carcass	108	0 - 112	7.69*	3.99
16	Subcutaneous fat area	60	16 - 104	17.57****	9.38
16	Subcutaneous fat depth	56	16 - 112	8.58*	4.55
19	Dressing percentage	88	0 - 96	5.57*	2.84
23	Carcass weight	40	4 - 80	6.86*	3.54
23	Final body weight	40	0 - 80	7.41*	3.85
23	Percent fat in carcass	12	0 - 80	6.22*	3.13
23	Percent lean in carcass	12	0 - 80	6.99*	3.60
23	Total fat	16	2 - 80	10.29**	5.49
23	Total lean	12	0 - 60	9.4**	4.99
24	Carcass bone	88	0 - 96	7.62*	3.98
26	Total lean	52	0 - 60	7.67*	4.00
